# Histone H3K4 Methyltransferase PeSet1 Regulates Colonization, Patulin Biosynthesis, and Stress Responses of *Penicillium expansum*

**DOI:** 10.1128/spectrum.03545-22

**Published:** 2023-01-12

**Authors:** Xiaodi Xu, Yong Chen, Boqiang Li, Shiping Tian

**Affiliations:** a Key Laboratory of Plant Resources, The Innovative Academy of Seed Design, Institute of Botany, Chinese Academy of Sciences, Beijing, China; b University of Chinese Academy of Sciences, Beijing, China; Universita degli Studi del Molise

**Keywords:** *Penicillium expansum*, H3K4 methyltransferase, Set1, blue mold, patulin biosynthesis, stress responses

## Abstract

Fruit blue mold disease and patulin contamination caused by *Penicillium expansum* lead to huge economic losses and food safety concerns worldwide. Many genes have been proven to be involved in the regulation of pathogenic and toxigenic processes of *P. expansum*. Histone H3 lysine 4 (H3K4) methylation is well recognized for its association with chromatin regulation and gene transcription. However, it is not clear whether H3K4 methylation is related to infection and patulin biosynthesis in *Penicillium*. Here, we characterized PeSet1, which is responsible for H3K4me1/me2/me3 in *P. expansum*. The deletion of *PeSet1* caused severe defects in hyphal growth, conidiation, colonization, patulin biosynthesis, and stress responses. Moreover, we demonstrated that PeSet1 is involved in the regulation of patulin biosynthesis by mediating the expression of patulin cluster genes and crucial global regulatory factors. Likewise, PeSet1 positively regulated key genes in β-1,3-glucan biosynthesis and the reactive oxygen species scavenging process to modulate cell wall integrity and oxidative stress responses, respectively. Collectively, we have proven for the first time the function of Set1 in patulin biosynthesis and the crucial role of Set1 in colonization and stress responses in *P. expansum*.

**IMPORTANCE** *Penicillium expansum* is one of the most important plant fungal pathogens, which not only causes blue mold rot in various fruits, leading to huge decay losses, but also produces mycotoxin patulin, posing a threat to human health. Both pathogenesis and patulin biosynthesis in *P. expansum* are regulated by complex and sophisticated networks. We focused on the epigenetic modification and identified a conserved histone H3K4 methyltransferase PeSet1 in *P. expansum*. Our work revealed the important role of PeSet1 in growth, development, colonization, patulin production, and stress responses of *P. expansum*. In particular, we originally described the regulation of Set1 on patulin biosynthetic pathway. These findings will provide new targets for the prevention and control of blue mold disease and patulin contamination.

## INTRODUCTION

*Penicillium expansum* is a harmful necrotrophic pathogen that causes blue mold rot on a variety of fruit worldwide during postharvest handling and storage (1, 2). Moreover, *P. expansum* is a major producer of mycotoxin patulin that contaminates apple juice and other derived fruit products (3). As patulin is acutely and chronically toxic to humans, long-term exposure and consumption of patulin-contaminated products can be extremely damaging to human health (4, 5). Nowadays, *P. expansum* has become a global concern for postharvest loss and food safety. An in-depth study of the molecular regulatory mechanisms of *P. expansum* pathogenesis and patulin biosynthesis will provide new ideas for control strategies. As is reported, both the infection and patulin biosynthesis process of *P. expansum* require the involvement of amounts of genes, forming a complex regulatory network (6, 7). In eukaryotic cells, regulation of gene expression is associated with covalent modifications of histones, such as methylation, acetylation, phosphorylation, ADP ribosylation, and ubiquitination (8, 9). The methylation of histone H3 lysine 4 (H3K4) is associated with active transcriptional activity and is highly conserved in a wide range of eukaryotic organisms (10).

Set1/MLL family of methyltransferases, catalyze the mono-, di-, or trimethylation of H3K4 (H3K4me1/me2/me3) by using their conserved SET (Suppressor of variegation 3–9 [Su(var)3-9], Enhancer of Zeste [E(z)], Trithorax) domain (11, 12). Humans harbor six H3K4 methyltransferses (MLL1-4, SETD1A, and SETD1B) and Drosophila melanogaster contains three (TRX, TRR, and SET1) (13). In yeast, only one methyltransferse Set1 is responsible for all three states of H3K4 methylation (10). Set1 is not active by itself but requires the presence of other components of COMPASS (complex proteins associated with Set1) for its full methyltransferase activity, including Bre2 (Cps60), Sdc1 (Cps25), Spp1 (Cps40), Swd1 (Cps50), Swd2 (Cps35), Swd3 (Cps30), and Shg1 (Cps15) (14–16). The crystal structure and conformational dynamics of the yeast COMPASS have been analyzed, which reveals the role of each subunit and the intricate coordination between them (17, 18).

In the last decade, the importance of Set1-mediated H3K4 methylation has been gradually revealed in fungi. Disruption of Set1 homologs causes a severe reduction in vegetative growth and/or conidiation of several crop fungal pathogens, including Magnaporthe oryzae (19), Fusarium graminearum (20), Fusarium fujikuroi (21), Fusarium verticillioides (22), Ustilaginoidea virens (23), and Aspergillus flavus (24). MoSET1 is involved in infection-related morphogenesis of M. oryzae, such as conidiation and appressorium formation (19). About 5% of M. oryzae genes change significantly in H3K4me2/3 abundance during infection-related morphogenesis, and H3K4me2/3 is associated with gene activation (19). COMPASS-like complex modulates the development and pathogenesis of M. oryzae by regulating H3K4me3-mediated targeted gene expression (25). Moreover, the SET1/KMT2-Cre1-Hyd pathway not only regulates the virulence of Beauveria bassiana and Metarhizium robertsii but also modulates the asexual cycle and stress responses of B. bassiana (26, 27). Similarly, deletion of *set1* in F. fujikuroi results in the loss of H3K4me2 and abolished GA_3_ production *in vitro*, which correlates with reduced virulence in rice (21). In Candida albicans, Set1 positively regulates the expression of mitochondrial protein genes through H3K4 methylation, thereby maintaining normal cellular reactive oxygen species (ROS) production and regulating fungal virulence (28).

For secondary metabolism and mycotoxin biosynthesis, H3K4 methylation mediated by Set1 is essential for the transcriptional activity of genes involved in deoxynivalenol (DON) and aurofusarin (red pigment) biosynthesis in F. graminearum (20). Deletion of *FvSet1* in F. verticillioides resulted in reduced fumonsins B1 (FB1) biosynthesis and downregulation of *FUM* genes (22). However, the H3K4 methylation status of these gene clusters remains to be determined. In addition, *F. fujikouroi* Set1 also regulated several secondary metabolite (SM) gene clusters located in subtelomeric regions and negatively affected production of bikaverin (BIK), fusarubins (FSR), fusarins (FUS), and fusaric acid (FSA) (21).

Despite the importance of Set1-mediated H3K4 methylation in many species, its role in *P. expansum* is not yet known. In this study, we identified the H3K4 methyltransferase PeSet1 in *P. expansum*. PeSet1 was responsible for H3K4me1/me2/me3 and was required for hyphae growth, development, colonization, and patulin biosynthesis of *P. expansum*. PeSet1 positively regulated patulin cluster genes, accompanied by the alteration of H3K4 methylation on specific gene regions. Moreover, PeSet1 modulated cell wall composition and oxidative stress response through transcriptional regulation of key genes. Taken together, we revealed the important regulatory role of PeSet1 in pathogenesis and patulin biosynthesis.

## RESULTS

### PeSet1 is responsible for H3K4 methylation in *P. expansum*.

**FIG 1:**
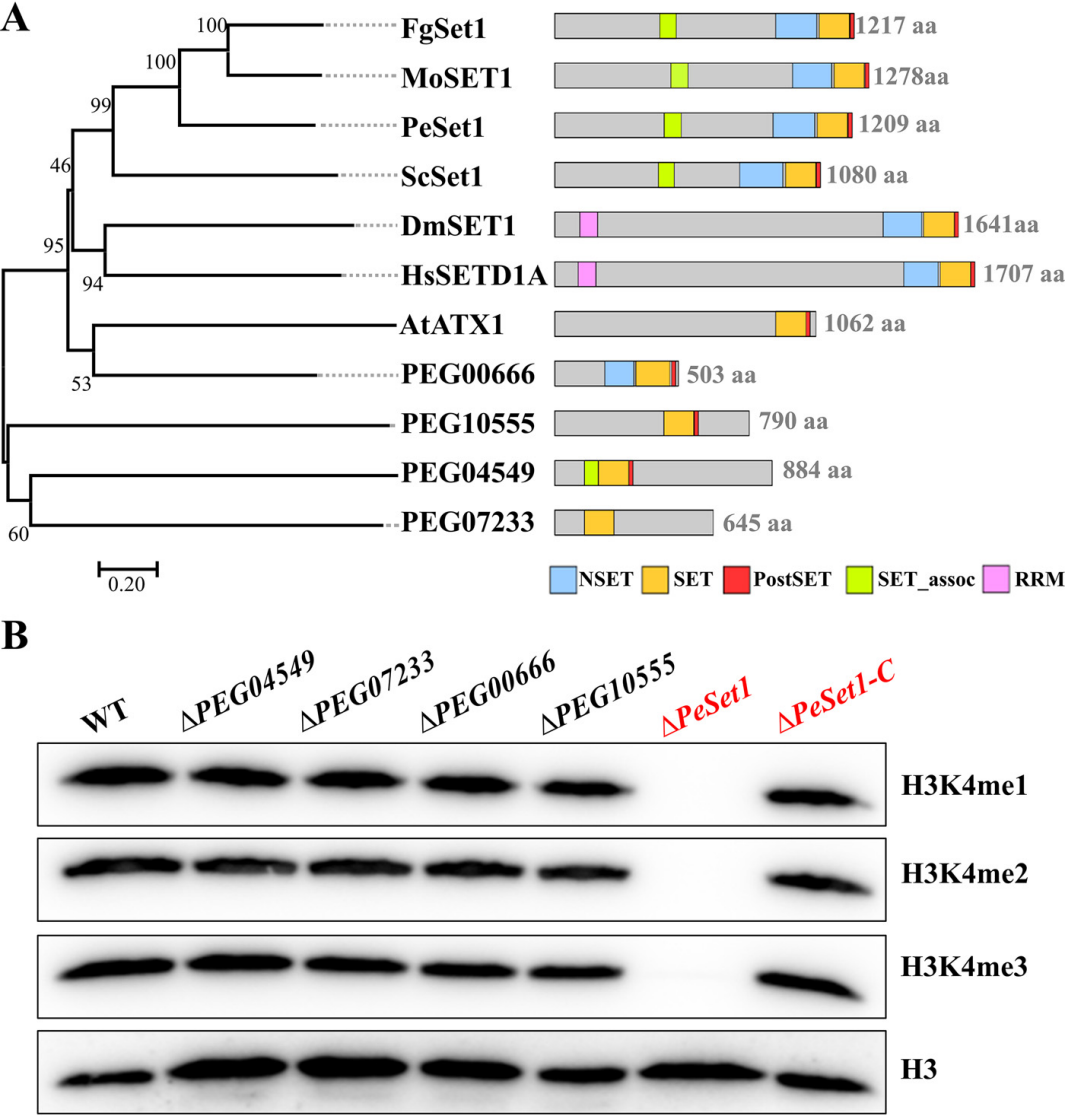
Identification of Set1 in *Penicillium expansum.* (A) Phylogenetic relationship and conserved domains of Set1 homologs in *P. expansum* (Pe), Saccharomyces cerevisiae (Sc), Fusarium graminearum (Fg), Magnaporthe oryzae (Mo), Drosophila melanogaster (Dm), Homo sapiens (Hs), and Arabidopsis thaliana (At), respectively. The phylogenetic tree was constructed with MEGA 11 using the neighbor-joining method. Conserved domains were predicted with SMART (http://smart.embl-heidelberg.de). (B) Western blot for mono-, di-, and trimethylated signals of H3K4 in deletion mutants of Set1 homologs in *P. expansum* and the complementation strain Δ*PeSet1-C*. RRM, RNA-recognition motif.

To identify Set1 homologs in *P. expansum*, the amino acid sequence of Saccharomyces cerevisiae Set1 (ScSet1) was used for BLASTp. Five proteins, PeSet1, PEG04549, PEG07233, PEG00666, and PEG10555, were obtained and PeSet1 shared the highest similarity (47.24%) with ScSet1 relative to the other four proteins. Phylogenetic relationships and conserved domains of Set1 homologs in seven species were further analyzed, including *P. expansum*, S. cerevisiae, F. graminearum, M. oryzae, D. melanogaster, Homo sapiens, and Arabidopsis thaliana. As shown in Fig. 1, PeSet1 is closely related to MoSET1, FgSet1, and ScSet1. All Set1 homologs possessed the conserved catalytic SET domain, and seven of them also contained the N-SET domain and PostSET domain, which are generally required for the catalytic activity of Set1 (29).

To determine whether Set1 homologs affect H3K4 methylation in *P. expansum*, *PeSet1*, *PEG04549*, *PEG07233*, *PEG00666*, and *PEG10555* were deleted, respectively. All the mutants were confirmed by PCR or Southern blot analyses as described in Materials and Methods. Histones of the mutants were extracted for detection of H3K4 methylation. The results showed that Δ*PeSet1* lost the global H3K4me1/me2/me3, which, moreover, was recovered in the *PeSet1* complementary (Δ*PeSet1-C*) strain (Fig. 1B). However, Δ*PEG04549,* Δ*PEG07233,* Δ*PEG00666,* and Δ*PEG10555* did not exhibit an obvious difference in the H3K4me1/me2/me3 levels in comparison with the wild-type (WT) strain. Taken together, PeSet1 is required for all three states of H3K4 methylation in *P. expansum*.

### PeSet1 is required for normal growth and development of *P. expansum*.

**FIG 2:**
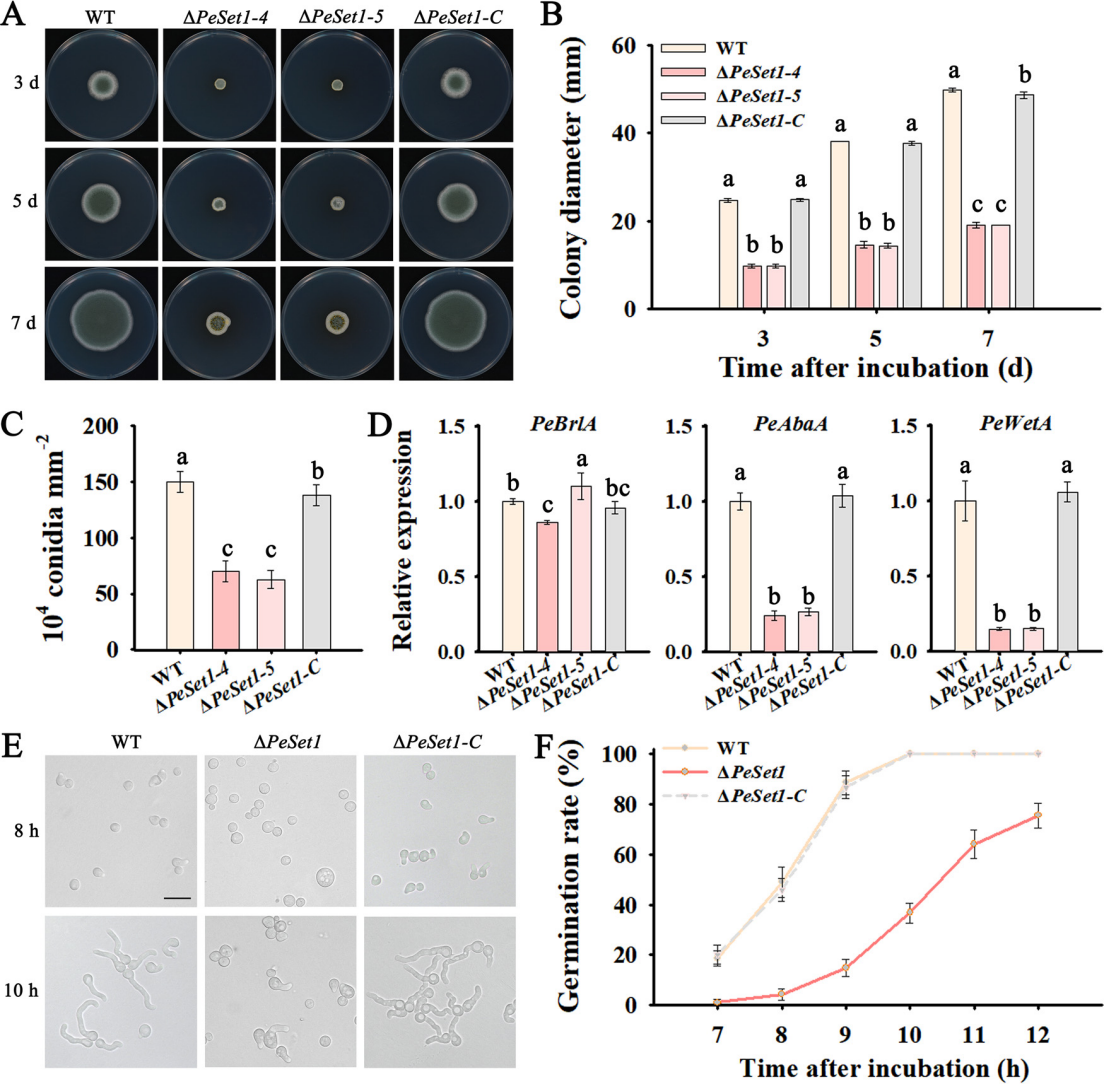
PeSet1 is required for growth and development of *P. expansum*. (A) Colony morphologies of the WT, Δ*PeSet1*, and Δ*PeSet1-C* strains on PDA after 3, 5, and 7 days of incubation. (B) Colony diameters of the strains after 3, 5, and 7 days of growth. (C) Conidiation of the strains after 12 days of incubation on PDA. (D) Expression analysis of the core regulatory genes in conidiation. (E) Morphologies of conidial germination of the WT, Δ*PeSet1*, and Δ*PeSet1-C* strains after 8 and 10 h of incubation on PDA. Bar = 20 μm. (F) Conidial germination rate of each strain. Columns with different letters are significantly different (*P < *0.05).

To further explore the role of PeSet1 in the growth and development of *P. expansum*, phenotype analysis of the WT, Δ*PeSet1*, and Δ*PeSet1-C* strains was conducted. Compared with the WT and Δ*PeSet1-C* strains, Δ*PeSet1* showed severe defects in growth and conidiation. The colony diameter of Δ*PeSet1* was reduced by about 61% compared with that of the WT strain at different stages of incubation (Fig. 2A and B). After 12 days of incubation, the conidia of Δ*PeSet1* were reduced by around 55% (Fig. 2C). Expression analysis of three key regulatory genes in the conidiation pathway revealed that *PeAbaA* and *PeWetA* were greatly downregulated in Δ*PeSet1*, indicating that PeSet1 positively regulates conidiation of *P. expansum* by mediating the two critical regulatory genes.

Conidial germination of *P. expansum* was also affected by PeSet1 (Fig. 2E and F). After 8 h of incubation, the germination rate of Δ*PeSet1* did not reach 10%, whereas that of the WT strain and Δ*PeSet1-C* was nearly 50%. While the WT strain and Δ*PeSet1-C* almost germinated completely after 10 h of incubation, the germination rate of Δ*PeSet1* was still less than 50%. The results indicated that PeSet1 is involved in conidial germination.

### PeSet1 is required for colonization of *P. expansum* on apple fruit.

**FIG 3:**
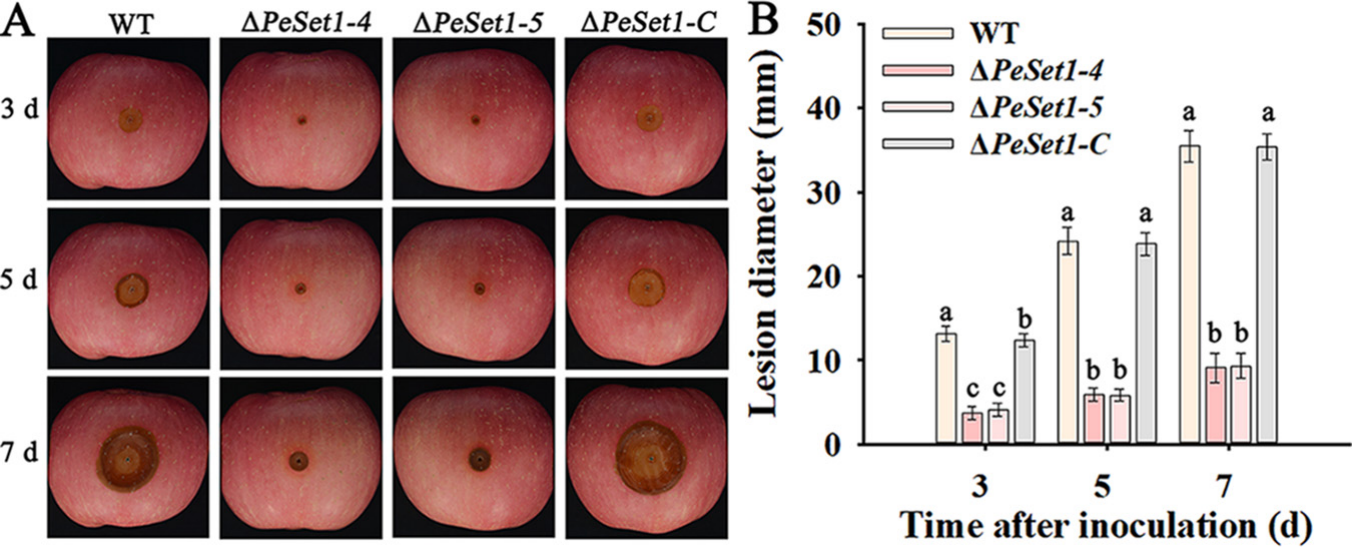
PeSet1 is important for the colonization of *P. expansum* on apple fruit. (A) Disease symptoms on apple fruit after 3, 5, and 7 days of inoculation. (B) Lesion diameters on apple fruit after 3, 5, and 7 days of inoculation. Columns with different letters are significantly different (*P < *0.05).

The role of PeSet1 in infection was further investigated. The WT, Δ*PeSet1*, and Δ*PeSet1-C* strains were inoculated on apple fruit, and lesions were detected daily. The disease symptoms of apple fruit infected by the WT and Δ*PeSet1-C* strains were severe, and the lesions reached almost 40 mm after 7 days of inoculation, whereas lesions of apple fruit infected by the Δ*PeSet1* mutant expanded slowly and were merely 10 mm (Fig. 3). The result indicated that PeSet1 is indispensable for colonization of *P. expansum* on apple fruit.

### PeSet1 regulates patulin biosynthesis in *P. expansum*.

**FIG 4:**
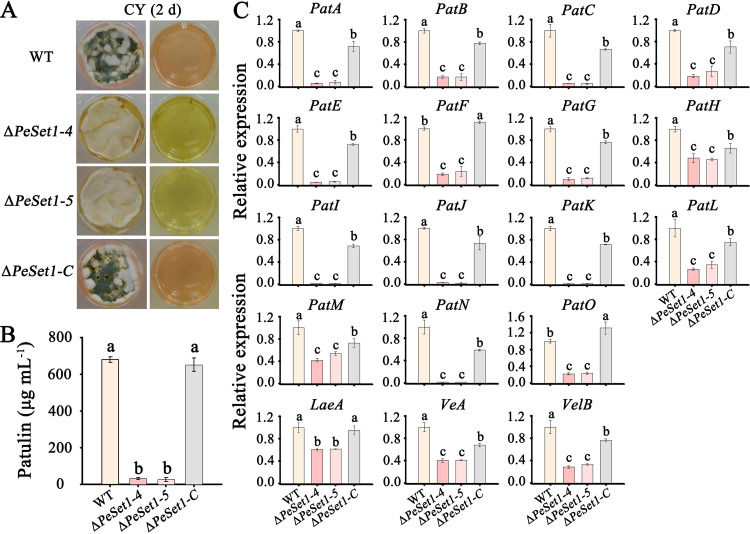
PeSet1 regulates patulin biosynthesis in *P. expansum*. (A) Colony morphologies and pigment biosynthesis of the WT, Δ*PeSet1*, and Δ*PeSet1-C* strains after 2 days of incubation on CY. (B) Patulin production of the indicated strains. (C) Expression analysis of 15 patulin cluster genes (*PatA*-*O*) and 3 regulatory genes (*LaeA*, *VeA*, and *VelB*) in the indicated strains. Columns with different letters are significantly different (*P < *0.05).

To explore whether PeSet1 has an impact on patulin biosynthesis, high-performance liquid chromatography (HPLC) analysis was performed to detect the patulin production. After 2 days of incubation on Czapek yeast extract (CY) medium, the content of patulin in Δ*PeSet1* decreased to about 4% of that in the WT and Δ*PeSet1-C* strains (Fig. 4B). Moreover, the color of CY medium-incubated Δ*PeSet1* was yellow but that of the WT and Δ*PeSet1-C* strains was reddish (Fig. 4A). These results indicated that PeSet1 is important for patulin as well as pigment biosynthesis.

The gene cluster responsible for patulin biosynthesis (*PatA*-*PatO*) has been characterized in *P. expansum*, which was found to be regulated by vital global regulators LaeA, VeA, and VelB (4, 5). To understand how PeSet1 affects patulin biosynthesis, expression levels of 15 patulin cluster genes (*PatA*-*PatO*) and 3 global regulatory genes (*LaeA*, *VeA*, and *VelB*) were analyzed in the WT, Δ*PeSet1*, and Δ*PeSet1-C* strains. All of the patulin cluster genes and global regulatory genes were downregulated in Δ*PeSet1*, while gene expression was almost recovered in Δ*PeSet1-C* (Fig. 4C). These results indicated that PeSet1 regulates patulin biosynthesis via regulating patulin cluster genes and key global regulatory genes.

**FIG 5:**
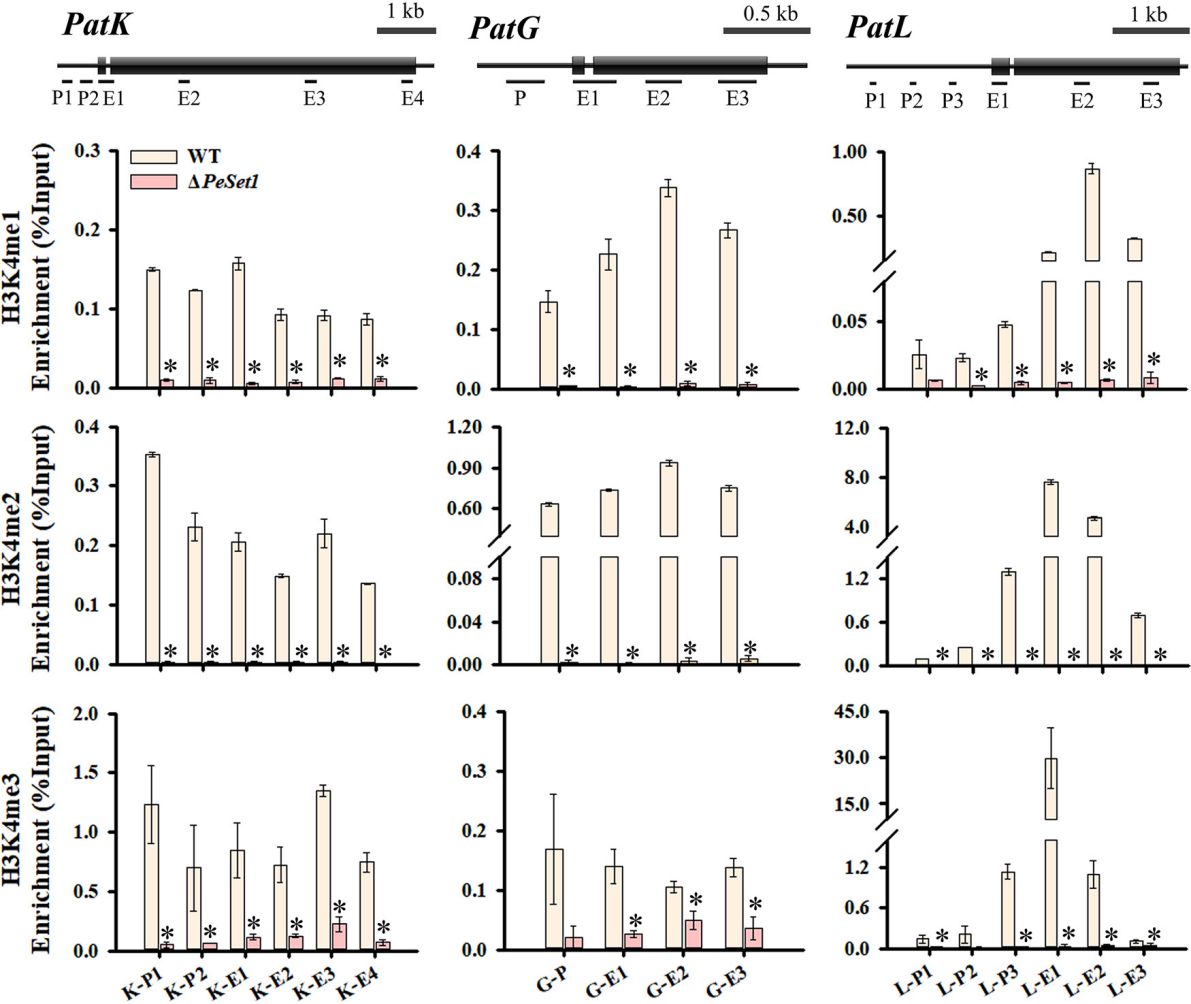
H3K4 methylation patterns in the specific regions of *PatK*, *PatG*, and *PatL*. ChIP followed by qPCR measurement of DNA enrichment is performed to analyze the H3K4 methylation patterns. Exons are indicated by boxes and introns are indicated by lines. P indicates the region in the promoter, and E indicates the region in the coding region. The relative enrichment of IP DNA was calculated by percentage of input. Asterisks indicate significant differences (*P < *0.05).

As PeSet1 is responsible for H3K4 methylation in *P. expansum*, the distribution of H3K4 methylation in specific regions of three key genes in the patulin cluster was analyzed by chromatin immunoprecipitation-quantitative PCR (ChIP-qPCR) assay (Fig. 5). *PatK* and *PatG* encode 6-methylsalicylic acid synthase and 6-methylsalicylic decarboxylase and are responsible for steps 1 and 2 reactions of patulin biosynthesis, respectively (7). *PatL* encodes a specific transcription factor of the patulin biosynthetic pathway. In Δ*PeSet1*, H3K4me1/me2/me3 levels were significantly reduced in all detection regions of *PatK*. H3K4me1/me2 levels were greatly reduced in all detection regions of *PatG* and H3K4me3 levels in coding regions *PatG*-E1-3. Similarly, H3K4me1/me2/me3 levels in most detection regions of *PatL* were also reduced, except for H3K4me1 in *PatL*-P1 and H3K4me3 in *PatL*-P2. As a whole, PeSet1 indeed affected H3K4 methylation at *PatK*, *PatG*, and *PatL*.

### PeSet1 regulates diverse stress responses of *P. expansum*.

**FIG 6:**
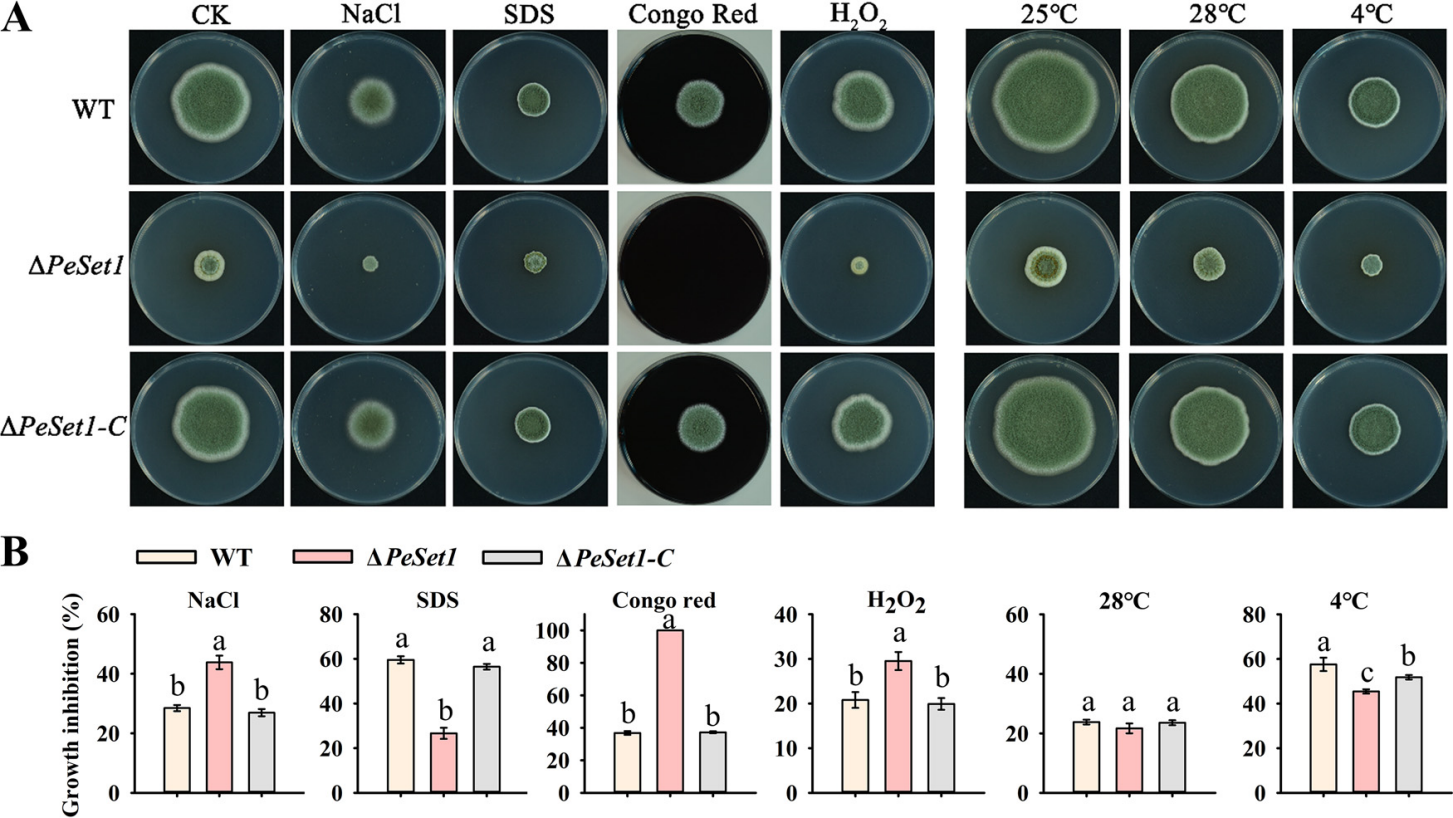
Roles of PeSet1 in stress responses of *P. expansum*. (A) Colony morphologies of the WT, Δ*PeSet1*, and Δ*PeSet1-C* strains after 7 days of stress treatment. NaCl (1.5 M), SDS (0.2 mg mL^−1^), Congo red (6.5 mg mL^−1^), and H_2_O_2_ (3 mM) were used to produce osmotic stress, membrane stress, cell wall stress, and oxidative stress, respectively. CK, control check. All strains were treated with heat (28°C) and cold (4°C) stress after 3 days of incubation on PDA at 25°C. (B) Growth inhibition rate of each strain after 7 days of stress treatment. Columns with different letters are significantly different (*P < *0.05).

**FIG 7:**
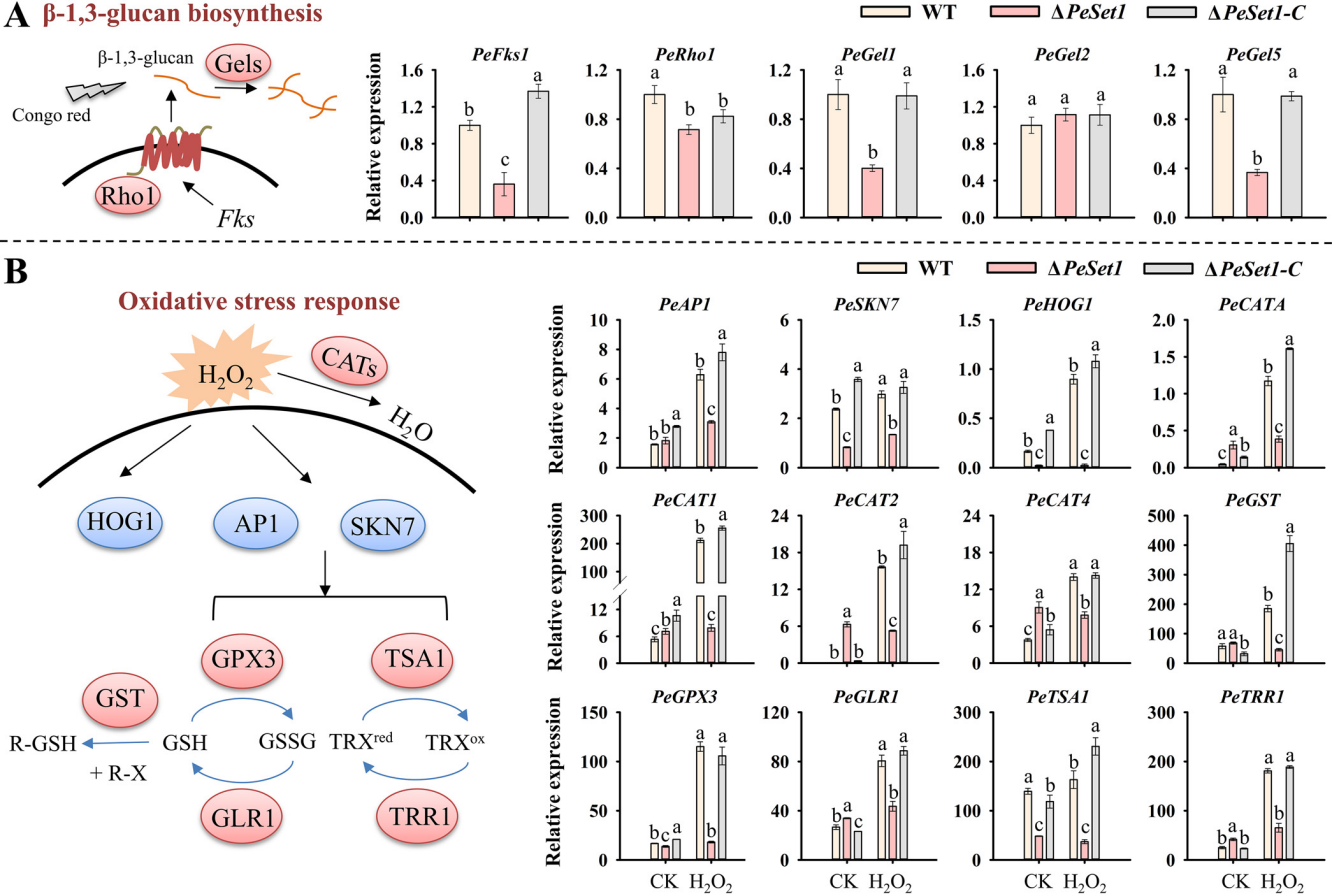
PeSet1 affects cell wall integrity (A) and regulates oxidative stress response (B). Relative expression levels in panel B are represented by 100-fold values after normalization with *β*-*tubulin*. CK means control check (1-day-old PDA culture without H_2_O_2_ treatment). H_2_O_2_ treatment of each strain was performed after 1 day of incubation on PDA for 4 h. Columns with different letters are significantly different (*P < *0.05). *Fks*, β-1,3-glucan synthase; Rho1, Rho GTPase; Gels, β-1,3-glucanosyltransferases Gel family; AP1, AP-1-like bZIP factor; SKN7, stress-responsive transcription factor; HOG1, high osmolarity glycerol response1; CATs, catalases; GST, glutathione *S*-transferase; GLR1, glutathione reductase; GPX3, glutathione peroxidase; TRR1, thioredoxin reductase; TSA1, thioredoxin peroxidase; GSH, reduced glutathione; GSSG, oxidized glutathione; TRX^red^, reduced thioredoxin; TRX^ox^, oxidized thioredoxin; R-X, xenobiotics.

The capacity of fungi to respond to stress affects its development and virulence, as well as their adaptation to the environment (30, 31). To investigate the effect of PeSet1 on stress responses, the WT, Δ*PeSet1*, and Δ*PeSet1-C* strains were subjected to osmotic stress (NaCl), membrane stress (SDS), cell wall stress (Congo red), oxidative stress (H_2_O*_2_*), and heat (28°C) and cold (4°C) stress. Δ*PeSet1* showed enhanced tolerance to SDS and 4°C, and reduced tolerance to Congo red, H_2_O*_2_*, and NaCl (Fig. 6). Especially, Δ*PeSet1* failed to grow under Congo red treatment, while the WT and Δ*PeSet1-C* stains showed less than 40% inhibition of growth (Fig. 6). Given that Congo red impedes cell wall assembly by specifically binding β-1,3-glucan in the cell wall, the transcript levels of key genes in β-1,3-glucan biosynthesis and remodeling were detected. Notably, the expression of *PeFks1*, *PeGel1,* and *PeGel5* was downregulated in Δ*PeSet1* compared to the WT and Δ*PeSet1-C* strains (Fig. 7A). *Fks1* and *Gel* family genes encode β-1,3-glucan synthase and remodeling enzymes, respectively, which are well known for its critical and conserved roles in cell wall integrity (CWI) of fungi (32, 33). Therefore, PeSet1 might be implicated in CWI by modulating the biosynthesis and structural remodeling of β-1,3-glucan.

In addition, transcript levels of genes encoding regulatory factors (AP1, SKN7, and HOG1) and detoxifying enzymes (CATA, CAT1, CAT2, CAT3, GST, GPX3, GRL1, TSA1, and TRR1) in oxidative stress response were detected. All 12 genes in the WT and Δ*PeSet1-C* strains were upregulated under H_2_O_2_ treatment (Fig. 7B), confirming the involvement of these genes in oxidative stress. Moreover, the deletion of PeSet1 resulted in the downregulation of these genes under H_2_O_2_ treatment (Fig. 7B), indicating the positive regulatory role of PeSet1 in oxidative stress response.

## DISCUSSION

H3K4 methylation by Set1/COMPASS is one of the important posttranslational modifications in histones and is associated with transcriptional regulation (12). Set1 is the catalytic center of Set1/COMPASS and is conserved enzymatically and structurally from yeast to humans (13). Among six Set1 homologs in humans, MLL1 and MLL3/4 are associated with the development of leukemia and cancer pathogenesis, respectively (13). The SET1 homolog SET2 is essential for life span and fertility in Caenorhabditis elegans (34). The plant homolog ATX1 (ARABIDOPSIS HOMOLOG OF TRITHORAX1) is involved in floral organ development, organogenesis, and responses to biotic and abiotic stresses (35–37). In fungi, Set1 is important for growth, development, secondary metabolism, and colonization on hosts, but its role in most postharvest pathogens, including P. expansum, remains uncertain.

In this study, we found that the absence of PeSet1 caused loss of H3K4me1/me2/me3 (Fig. 1) and severe defects in growth, conidiation, conidial germination, and colonization on apple fruit (Fig. 2 and 3). However, the other four Set1 homologs in *P. expansum* had no or slight impact on H3K4 methylation and fungal growth and colonization (Fig. S2, S3, and S4 in the supplemental material). As for other fungal pathogens, the *Moset1* or *Mrkmt2* deletion mutants exhibited severe defects in hyphal growth, conidiation, appressorium formation, and virulence of M. oryzae and *M. robertsii*, respectively (19, 26). In F. graminearum and *B.* bassiana, Set1 is also required for hyphal growth and virulence and functions in conidiation and conidial quality control of B. bassiana (20, 27). *P. expansum* infects host cells dependent on conidia and invasive hyphae without other special invasive structures. Thus, impaired colonization of Δ*PeSet1* may be partially due to defects in conidiation, germination, and hyphal growth. In fungal conidiation, *BrlA*, *AbaA*, and *WetA*, core regulatory genes, are responsible for conidiation initiation, phialides differentiation, and spore formation and maturation, respectively (38–41). In B. bassiana, *BrlA* and *AbaA* were dramatically downregulated and *WetA* was less suppressed in Δ*set1* relative to the WT strain (27). Similar to these findings, our data showed that disruption of *PeSet1* in *P. expansum* led to severely decreased expression of *PeAbaA* and *PeWetA*, suggesting that PeSet1 is involved in the regulation of conidiation.

The stress responses of fungi are critical to their survival and invasion (30). ROS are highly damaging to cells. Fungal pathogens are exposed to ROS that are generated by the host plant or animal as a major defense mechanism (42). To reduce oxidative damage, a set of responsive genes were induced in pathogens to encode oxidative stress detoxification and repair proteins, including catalases (CATs), glutathione *S*-transferase (GST), glutathione reductase (GLR1), glutathione peroxidase (GPX3), thioredoxin reductase (TRR1), and thioredoxin peroxidase (TSA1) (31, 43). The AP1-like bZIP factor AP1 and the response regulator SKN7 regulate oxidative stress-responsive genes in a collaborative manner (44, 45). HOG1 stress-activated protein kinase (SAPK) coordinates the response to osmotic stress and plays a role in oxidative stress tolerance (46, 47). Our results found that PeSet1 not only regulated a set of oxidative-responsive genes but also affected transcript levels of vital response regulators. It has been reported that Set1 contributed resistance to external ROS generated by the host of C. albicans (28). Thus, PeSet1-mediated transcriptional regulation may also function in the defense response of *P. expansum* against ROS stress from its hosts.

PeSet1 is also correlated with CWI as the increased sensitivity of Δ*PeSet1* to Congo red (binding β-1,3-glucan). In fungi, β-1,3-glucan synthase (encoded by the *Fks* genes) and the Rho GTPase Rho1 form the β-1,3-glucan synthase complex (48, 49). The Gel family of remodeling enzymes reconstitutes and cross-links the sugar chains (50). Aspergillus fumigatus owns seven *Gel* genes (*Gel1-7*) (51). *P. expansum* possesses six *Gel* homologs (*PeGel1-6*), of which *PeGel1*, *PeGel2*, and *PeGel5* were normally expressed in the WT strain. Our results determined that PeSet1 positively regulated expressions of *PeFks1*, *PeGel1*, and *PeGel5*. Likewise, B. bassiana Set1 modulated genes involved in cell wall composition (β-1,3-glucan and chitin) and CWI pathway (Slt2-Mkk1-Bck1 cascade) (27). In contrast to the hypersensitivity of Δ*PeSet1* to Congo red, deletion of *Set1* in F. graminearum and F. verticillioides reduced sensitivity to Congo red, suggesting that Set1 modulates fungal CWI in diverse regulatory manners (20, 22). Taken together, PeSet1 may mediate the expression of *PeFks1*, *PeGel1*, and *PeGel5* to modulate β-1,3-glucan biosynthesis and structure remodeling, thereby contributing to CWI and fungal cell survival.

Genes responsible for SM biosynthesis are often contiguously clustered and easily affected by chromatin modifications (52). Set1 and H3K4 methylation have been shown to regulate biosynthesis of various SMs, including mycotoxins, pigments, phytohormones, as well as unidentified SMs (21, 22, 53, 54). We found Set1 played a critical role in patulin production for the first time and proposed that PeSet1 may serve as a positive regulator of patulin cluster genes. Our results are consistent with the role of Set1 in the regulation of mycotoxin biosynthesis in *F graminearum* and A. flavus (20, 24). Set1-mediated H3K4 methylation is associated with active chromatin in diverse eukaryotic organisms. In particular, H3K4me3 is widely recognized as a marker of active transcription to a larger degree than H3K4me1/me2 and mainly enriches at the promoter and transcriptional start site (55–57). In *U. virens*, ChIP-seq and RNA-seq assays revealed that H3K4me3 participates in transcriptional activation of conidiation-related and pathogenic genes (23). Similarly, the expression of COMPASS target genes is highly correlated with H3K4me3 at transcription start sites in M. oryzae (25). In S. cerevisiae and F. graminearum, H3K4me3 or Set1 is associated with the RNA polymerase II (Pol II) complex during transcription initiation (20, 58). Our results revealed that H3K4me3 enriched at E1 regions and P regions near E1 of *PatL*, *PatK*, and *PatG*. Meanwhile, the absence of PeSet1 led to downregulation of *PatL*, *PatK*, and *PatG* accompanied by reduced H3K4me3 levels at these genes (Fig. 5), suggesting that PeSet1 could enhance transcription of patulin cluster genes by improving the H3K4me3 levels at the promoter and coding region near the transcriptional start site. Correspondingly, the deletion of *PeSet1* reduced H3K4me3 levels at specific regions of *PatL*, *PatK*, and *PatG* and decreased expression of these genes. To comprehensively understand how the distribution of H3K4 methylation is dynamically regulated and influences gene expression in secondary metabolism, a global scale of ChIP-seq analysis of H3K4me3 and PeSet1 along with RNA-seq analysis of Δ*PeSet1* will be carried out in the future study. Furthermore, the global transcription factor LaeA and the velvet family regulatory proteins VeA and VelB are well known for their crucial roles in regulating secondary metabolism in filamentous fungi (59). In *P. expansum*, LaeA, VeA, and VelB can affect patulin biosynthesis by regulating the expression of 15 patulin cluster genes (4, 5, 60). We found that PeSet1, as a writer of H3K4 methylation, regulated not only 15 genes (*PatA*-*PatO*) in the patulin cluster but also LaeA, VeA, and VelB, suggesting that PeSet1 contributes to patulin cluster gene expression partially by the three global transcription factors. Taken together, PeSet1 may regulate the patulin biosynthetic process via H3K4me3 as well as global transcription factors.

As a whole, PeSet1 plays a crucial role in a variety of biological processes, including growth, development, colonization, patulin production, and stress responses in *P. expansum*. These results may provide a new sight for controlling blue mold disease. As H3K4 methyltransferases play a significant role in the development of various cancers and drug resistance in human, potent inhibitors targeting H3K4 methyltransferase are currently under development for cancer treatment (61, 62). Therefore, PeSet1 may be utilized as a potential target to screen chemical compounds or biological molecules, which can block gene expression or inhibit enzyme activity/stability of PeSet1 and eventually control the infection of *P. expansum* and patulin contamination.

## MATERIALS AND METHODS

### Fungal strains and culture conditions.

*P. expansum* T01 was used as the WT strain in this study (63). The whole-genome sequence data of *P. expansum* T01 (accession number GWHBOZT00000000) have been deposited in the Genome Warehouse in National Genomics Data Center (64) and are publicly accessible at https://ngdc.cncb.ac.cn/gwh. All strains were cultured on potato dextrose agar (PDA) at 25°C in the dark. The conidia were collected using 0.05% Tween 20 and four layers of sterile gauze before being counted by an automatic cell counter (IY1200; Countstar).

### Phylogenetic relationships and conserved domain analysis.

Homologs of S. cerevisiae Set1 (NP_011987.1) in *P. expansum* were obtained by BLASTp, including PeSet1 (PEG09159, GWHGBOZT002811), PEG04549 (GWHGBOZT005499), PEG07233 (GWHGBOZT000901), PEG00666 (GWHGBOZT010252) and PEG10555 (GWHGBOZT008106). Set1 in other five species were searched by NCBI (http://www.ncbi.nlm.nih.gov/), including F. graminearum FgSet1 (XP_011327217.1), M. oryzae MoSET1 (MGG_ 15053T0), A. thaliana ATX1 (NP_850170.1), D. melanogaster SET1 (NP_001163851.1), and H. sapiens SETD1A (NP_055527.1). The multiple sequence alignment of all the above proteins was performed by Clustal W. A neighbor joining (NJ) tree was constructed by MEGA 11 software with 1,000 bootstrap replications. The conserved domains of the proteins were analyzed by SMART (http://smart.embl-heidelberg.de).

### Gene deletion and complementation.

For gene deletion, 5′-flank and 3′-flank sequences (about 1 kb) of the target gene were cloned into the vector pCHPH containing the hygromycin phosphotransferase gene *hph*. *Agrobacterium*-mediated transformation and the gene replacement strategy were carried out as previously mentioned (63). Transformants were chosen using 250 μg mL^−1^ hygromycin B. Then, the transformants were identified by PCR and further confirmed by Southern blot (Fig. S1). For gene complementation, the full length of the gene was cloned into the vector pCNEO harboring a selective gene *neo*, and transformants were selected by 250 μg mL^−1^ G418 and identified by PCR. All primers used for PCR are listed in Table S1.

### Phenotype analysis.

Phenotype analysis was performed as previously described (63, 65, 66), and all the experiments were repeated three times.

For growth tests, a 5-μL droplet of conidial suspension (1 × 10^5^ conidia mL^−1^) was incubated on each PDA plate and cultured at 25°C. The colony diameters were measured after 3 days, 5 days, and 7 days of incubation and then photographed.

For conidiation, conidia were harvested after 12 days of incubation on PDA and counted after filtration. Two-day-old mycelia were harvested from PDA for expression analysis of conidiation regulatory genes.

For conidial germination, aliquots of a conidial suspension (3 × 10^7^ conidia mL^−1^) were spread on PDA plates with cellophane sheets. The germination rate was examined with a microscope (Leica DM2000, Germany) after 8 to 11 h of incubation. In each strain, approximately 200 conidia were randomly observed.

For infection test, four wounds were evenly spaced across the equator of each apple fruit. The conidial suspension (1 × 10^5^ conidia mL^−1^) was pipetted into each wound in 5-μL. For every replication, there were at least six apple fruit for each strain. The inoculated apples were kept in plastic trays at 25°C for 7 days.

For patulin biosynthesis analysis, aliquots of a 1-μL conidial suspension (1 × 10^6^ conidia mL^−1^) were spread on PDA plates with 1 × 1-cm cellophane sheets. After 1 day of incubation, mycelia along with cellophane sheets were transferred and floated on 1 mL CY medium. After 2 days of culture without shaking in the dark, the mycelia were collected for RNA isolation and the CY medium was filtered via 0.45-μm filters for HPLC assay. In an isocratic elution mode, the mobile phase was a combination of water and acetonitrile flowed at a rate of 1 mL min^−1^ (90:10, vol/vol). A wavelength of 276 nm was used for patulin detection.

### Stress tolerance tests.

For heat and cold stress treatments, aliquots of 5-μL conidial suspensions (1 × 10^5^ conidia mL^−1^) were incubated on PDA at 25°C for 3 days and then incubated at 28°C and 4°C for 7 days. For other environmental stress treatments, strains were incubated on PDA plates with 1.5 M NaCl (osmotic stress mediator), 6.5 mg mL^−1^ Congo red (cell wall inhibitor), 0.2 mg mL^−1^ SDS (membrane-damaging agent), and 3 mM H_2_O_2_ (oxidative stress mediator), respectively, and kept for 7 days at 25°C. The experiments were repeated three times.

### Reverse transcription and quantitative PCR analysis.

Total RNA was isolated from mycelia using TRNzol Universal Reagent (Tiangen; DP424) according to the instructions. Reverse transcription and quantitative PCR (qPCR) were carried out as described previously (63, 66). The quantitation of gene expression was normalized by *β*-*tubulin* gene and relative expression levels were calculated with the 2^−ΔΔCt^ method (67). For relative enrichment of H3K4me1/me2/me3 at specific regions of *PatK*, *PatG*, and *PatL*, the values were calculated by dividing the amount of ChIP DNA by the input DNA. The detection regions included the regions in the promoter (P) and the coding region (E). The primers used for qPCR are displayed in Table S2.

### Histone extraction and Western blot.

Histone extraction was modified from the method previously described (68). Fresh mycelia were ground with liquid nitrogen and homogenized in 10 mL extraction buffer (10 mM Tris-HCl pH 7.5, 2 mM EDTA, 0.25 M HCl, 5 mM DTT, and 1× protease inhibitor cocktail). The fragments were removed by filtration with one layer of Miracloth (Calbiochem). The supernatants were separated after centrifugation (12,000 × *g*, 10 min) and 20% trichloroacetic acid was added to it to precipitate soluble proteins. The pellets were collected after centrifugation (17,000 × *g*, 30 min) and washed twice with ice-cold acetone. Histones were resuspended in lysis buffer (7 M urea, 2 M thiourea, 2% carrier ampholytes, 4% CHAPS, and 1% DTT) and quantified by the Bradford method. The procedure of Western bolt was performed as previously described with a few modifications (66). An equal amount of histone (10 μg) was separated by 15% SDS-PAGE. Primary antibodies used in this study include anti-Histone H3 (Abcam; ab1791) and anti-H3K4me1 (Abcam; ab8895), anti-H3K4me2 (Abcam; ab7766), and anti-H3K4me3 (Abcam; ab8580).

### ChIP assay.

ChIP assay was performed according to the method previously described (20, 69) with some modifications. After being ground by liquid nitrogen, mycelial powder was resuspended with 10 mL of ChIP extraction buffer I (0.4 M sucrose, 10 mM Tris-HCl pH 8.0, 10 mM MgCl_2_, 1 mM DTT, 0.1 mM PMSF, and 1 × protease inhibitor cocktail). The grounds were fixed with 1% formaldehyde in ChIP extraction buffer I and rotated for 10 min at 4°C. A final concentration of 0.125 M glycine was used to stop the cross-linking reaction for 5 min. Then, the grounds were filtered through Miracloth (Calbiochem) and centrifuged at 4°C. Then, the supernatants were removed and the precipitates were suspended in ChIP extraction buffer II (1.7 M sucrose, 10 mM Tris-HCl pH 8.0, 1% Triton X-100, 10 mM MgCl_2_, 1 mM DTT, 0.1 mM PMSF, and 1× protease inhibitor cocktail). After centrifugation, the nuclei (precipitates) were resuspended in nuclear lysis buffer (50 mM Tris–HCl pH 8.0, 1% SDS, 10 mM EDTA, 1 mM DTT, 0.1 mM PMSF, and 1× protease inhibitor cocktail) and kept on ice for 30 min. The chromatin was sheared by sonication (10 s on, 60 s off, for 4 times, amplitude 30%) to an average size of 0.5 kb of the DNA fragments. After centrifugation, the supernatants were diluted with nine volumes of ChIP dilution buffer (16.7 mM Tris–HCl pH 8.0, 1.1% Triton X-100, 1.2 mM EDTA, and 167 mM NaCl). A small aliquot of supernatants was set aside as the input and the remaining was incubated with antibodies and Dynabeads Protein G (Life Technologies; 10003D). Specific antibodies for H3K4 methylation included anti-H3K4me1 (Abcam; ab8895), anti-H3K4me2 (Abcam; ab7766), anti-H3K4me3 (Abcam; ab8580), and rabbit IgG (Abcam; ab150077) were used as control. After wash, elution, and reversed cross-link, the immunoprecipitated DNA was extracted by a TIANquick Midi purification kit (Tiangen; DP204) and analyzed by qPCR.

### Statistical analysis.

Data were analyzed by SPSS software (SPSS Inc., Chicago, IL, USA). Differences among multiple groups of means were analyzed by Duncan’s multiple range test. For comparisons in H3K4 methylation enrichment, a Student's *t* test was used to determine the differences between two groups of means. Differences at *P < *0.05 were considered statistically significant.
