# Supplementary material for: Histone H3K4 Methyltransferase PeSet1 Regulates Colonization, Patulin Biosynthesis, and Stress Responses of Penicillium expansum
Source: Microbiol Spectr. 2023 Jan 12;11(1):e03545-22. doi: 10.1128/spectrum.03545-22 (PMC9927251; doi:10.1128/spectrum.03545-22)
Supplement: Supplemental file 1 — Fig. S1 to S4 and Tables S1 and S2. Download spectrum.03545-22-s0001.pdf, PDF file, 0.7 MB [file spectrum.03545-22-s0001.pdf]

Supplementary materials

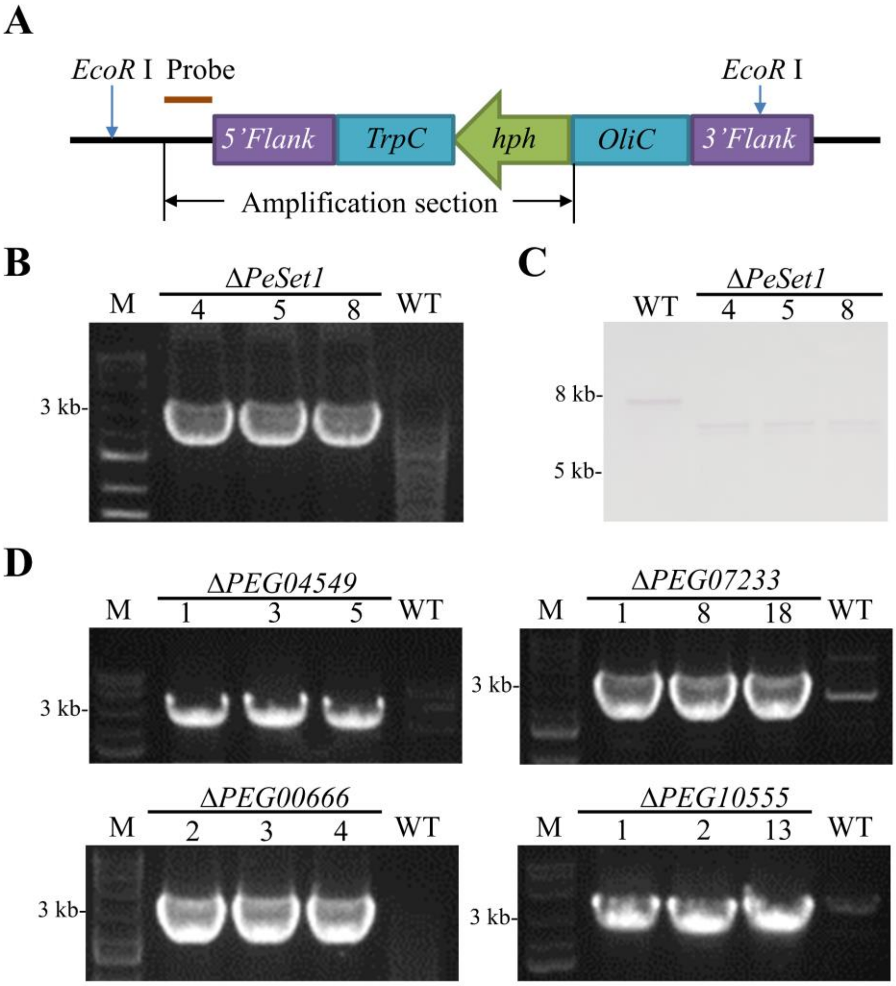

Figure S1. Identification of Δ*PeSet1*, Δ*PEG04549*, Δ*PEG07233*, Δ*PEG00666*, and Δ*PEG10555*. (A) The amplification section of PCR and the probe for Southern blot analysis. (B) PCR identification of Δ*PeSet1*. (C)Southern blot analysis of Δ*PeSet1*. The bands in the WT strain and Δ*PeSet1* were 7.61 kb and 6.33 kb, respectively. (D) PCR identification of Δ*PEG04549*, Δ*PEG07233*, Δ*PEG00666*, and Δ*PEG10555*.

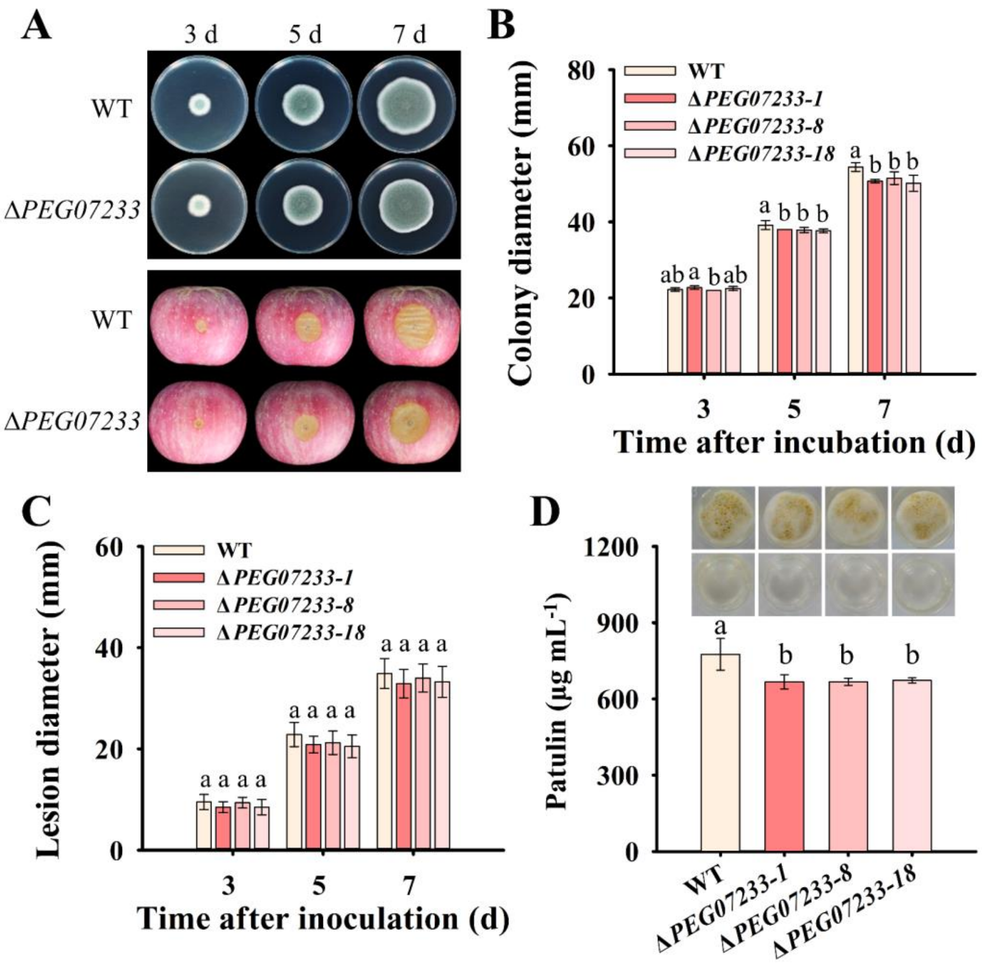

Figure S2. Phenotypic analysis of Δ*PEG07233*. **(**A) Colony morphologies of the WT and Δ*PEG07233* strains on PDA and disease symptoms on apple fruits after inoculating with each strain. (B) Colony diameters of the WT and Δ*PEG07233* strains after 3, 5, and 7 d of incubation on PDA. (C) Lesion diameters on apple fruits after 3, 5, and 7 d of inoculation. (D) Patulin production of the WT and Δ*PEG07233* strains after 2 d of incubation on CY. Columns with different letters represent significant differences (P < 0.05).

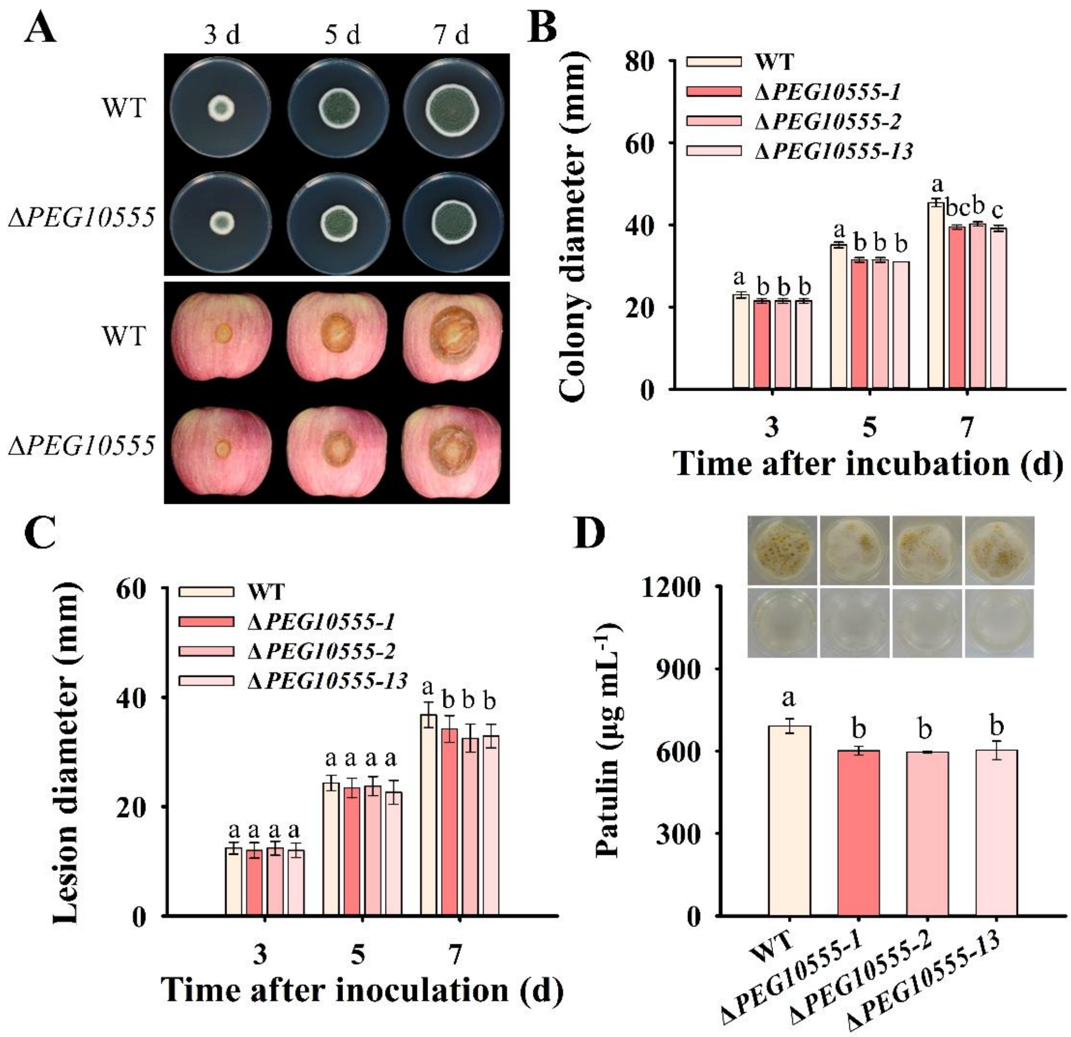

Figure S3. Phenotypic analysis of Δ*PEG10555*. (A) Colony morphologies of the WT and Δ*PEG10555* strains on PDA and disease symptoms on apple fruits after inoculating with the indicated strains. (B) Colony diameters of the WT and Δ*PEG10555* strains after 3, 5, and 7 d of incubation on PDA. (C) Lesion diameters on apple fruits after 3, 5, and 7 d of inoculation. (D) Patulin production of the WT and Δ*PEG10555* strains after 2 d incubation on CY. Columns with different letters represent significant differences ($P < 0.05$).

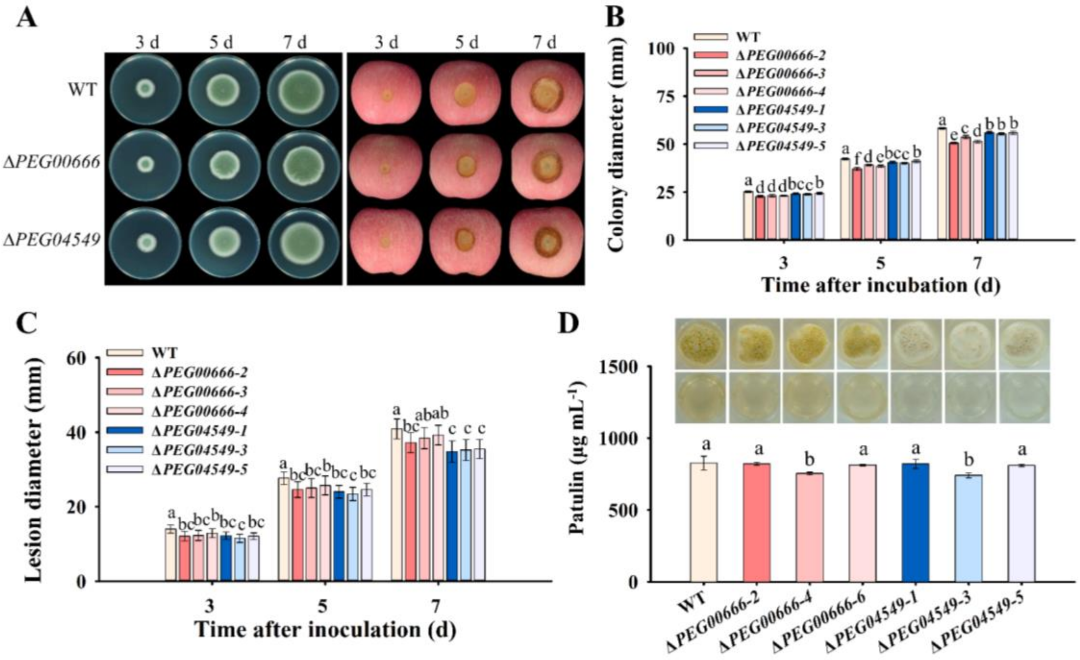

Figure S4. Phenotypic analysis of Δ*PEG00666* and Δ*PEG04549*. (A) Colony morphologies of the WT, Δ*PEG00666*, and Δ*PEG04549* strains on PDA plates and disease symptoms on apple fruits after inoculating with the indicated strains. (B) Colony diameters of the indicated strains after 3, 5, and 7 d of incubation on PDA. (C) Lesion diameters on apple fruits after 3, 5, and 7 d of inoculation. (D) Patulin production of the indicated strains after 2 d incubation on CY. Columns with different letters represent significant differences ($P < 0.05$).

Table S1. The primers used for construction and identification of gene deletion mutants and complementation strains.

**Name**	**Primer sequence (5'-3')**	
PeSet1-up	F:	CAAGCTTGATCCAACCGGTCTAGCGTGAC
	R:	AGGCGCGCCTGGTGAAAGGTTGGCTACACGC
PeSet1-down	F:	CGGATCCGAACCGCTTTCCAAGATCTTTCT
	R:	CGAGCTCGCACCGACGTTGTCCATTATCC
PEG04549-up	F:	CAAGCTTGGTGCAGCCATGAGGTTCAAGTTTG
	R:	GGCGCGCCGACAACACGGACTACGATTGGGAG
PEG04549-down	F:	CGGATCCGGCAACTCCAGCGGCTCTTGTCTT
	R:	GGAATTCCGAGCGTGAAACCGAGGAGATGTG
PEG07233-up	F:	CAAGCTTGATCAGCCATGGTACGTCCAAATC
	R:	GGCGCGCCGGTGGACGTAGCAAGAAGCTGAT
PEG07233-down	F:	CGGATCCGACAGTACAGCCCTGGAGCAATG
	R:	GGAATTCCTAGGAAGCCGAAGGCATTGAC
PEG00666-up	F:	TTAATTAATAAAATAGGCGAACTCCGATGC
	R:	AGGCGCGCCTAAGCAGCACGTGGGTGTTAGG
PEG00666-down	F:	GGGTACCCTATGCTTTCCTTTATTTCAACCATG
	R:	CGAGCTCGGGGGCCCTAATTAGGTTACAGATAT
PEG10555-up	F:	GAGCTCCACATAAGTTTGCCAAGACTGAC
	R:	GGATCCTTTCGAGAGAATGAGATAACAAGA
PEG10555-down	F:	GGCGCGCCTTAACCTCTGGTCAAACTACAGTCA
	R:	AAGCTTTCACTGGTGTCGATGATCCTTAT
PeSet1-geno	F:	GTCCGCAAATGTTGATTCTGTT
PEG04549-geno	F:	CCAAGGGACAGTTGAGGAAGCG
PEG07233-geno	F:	GCAGGAACATCTACTCGAGCC
PEG00666-geno	F:	TTCATTTTGTATTAGGGCGCG
PEG10555-geno	F:	CCATCTTCCTGACCGGTTACAT
hph	F:	TCACCCCCATCTCAACTCCA
	R:	TGCTCCATACAAGCCAACCAC
Com-*PeSet1*	F:	TAAAACGACGGCCAGTGCCAAGCTTATCCAACCGGTCTAG CGTGAC
	R:	CATGCATGGTTGCCTAACTAGGCGCGCCCACCGACGTTGT CCATTATCC

Table S2. The primers used for qPCR in this study.

**Name**	**Forward primer (5'-3')**	**Reverse primer (5'-3')**
PatA	AAAGGCCGGTGCATTGATC	TTGGAGGCTTTGGTGAGCAT
PatB	GCCAGGCTATGCGATTGAGT	GCTGGAACCCTGTCCATTGT
PatC	TCCACCTGCGAATATCCCTTA	CATCGCCAGTGCCATTTTC
PatD	ATGAGATTCGTCTGCGCAAAG	CTACCCAAGCGGGATGAGATT
PatE	CATTCTCATCGGGCCTGAGT	TCGAAGCTCTTCCGGACATG
PatF	GCGAGTGAATTCGGCCAAT	GTCCGACCCAAAGGATGAAG
PatG	CGGCCGTCTTGAAGGAAAT	CTTGCCGTAGCGGGTGAATA
PatH	CATTTATCGGCGGTGTTCTGA	GATCAACGCTTGCACGATAGC
PatI	GCAAACTCATTCCGCAAGGA	TGGTTCTTGCCATCGATCAC
PatJ	CGCCAGACATACCGCCATA	TTTGGTCGATCGGGACTGTT
PatK	GACGCTGGGCTACTGGATTG	TCGTGCGTGAGGCCAGTAT
PatL	GCAGGAGATCCGTTTCAGACA	CCACTGACCGACGGTTACAAC
PatM	ACCCACAGCTGCACATGGA	AGCGAGAAGAGGCGGAAGA
PatN	CGTTCGATGTCGCTAGCAAA	GGCGATAATCACGTCAATTCG
PatO	TCGCCTCCTGGTGTGTATCTT	AAGCGTGCCCAGTCATTCAG
LaeA	TCGAAGCTCTTCCGGACATG	AATGCAATGCGGTCAATCTG
VeA	GAAGATACTTTCGGCCTTGATGA	TCGCGATAAGCAGGATAAGGA
VelB	TTGTTGTCTGGGTCCGTCAA	CCTGGGATGAGGGATTCGT
PeBrlA	CGTGACCCCTCCTTCTTCTG	TCGGTGTGGAGTAGAAGGAGTGT
PeAbaA	CGACCACACCCGTCACTTCT	CGACAGCCGGTGAGAGCTA
PeWetA	CATCGCCGAATATTGCAATG	GGGCTGGAGTTGGTCAAGGT
PeFks1	GGCCAAGCACTTCGGTTCT	AAGTTCTGGTGCACGGCATT
PeRho1	CTGTGGACTCCCCCGATTC	ATAGGGAGACCCTGGCAGAAG
PeGel1	ACCCCCAACAAGGATTACGAT	AGAGTTGTAGCCGCCGTTTC
PeGel2	CACCCAGGCCAATGTTAAGG	GCAGATCCCAGGCGTTGTAG
PeGel5	TACTCGTGGTGCGGCAATT	GGGACGGAGTAGTTGGCAAA
PeAP1	CATCAGAATGGTGGCCAGTTC	AGCCGAAATCCTGAGACAACA
PeSKN7	TGAGGACGATGCCACTTGTC	CCGTCAAACGCGGTATCAAT
PeHOG1	CCGCATCCAAGAACCACATA	TTCGCCATGTGAGCATGATC
PeCATA	TGCTAATGAGGCTGTGCCATT	TGAGCGGAGAACCACTTTGC
PeCAT1	ACGCTCCACACCCTTCTTTG	TGCACACCGAATCCATCAAC
PeCAT2	ACGACGCCATGGATGAGAAC	GAAAGTCTCCACGCTCGATAGC
PeCAT4	CGCGGTACCCCATACTCCTA	AAGGTGCCATCTGGCTTGAT

PeGST	CTAAGCCGGAGCATGGTCTT	CTCAGCCCAACGCTTAAACC
PeGPX3	GGCTCGGACGATGATATCCA	GCGCCATTCACATCCAGTTT
PeGLR1	CTGCGCAAATTCGACCCTAT	TGGTTGCGGTGAAGGTTGA
PeTSA1	TCATCGACCCTGCCAAGAAG	CCGGAGAACCTCTGCTGTGT
PeTRR1	GCCCGGTACCAGCTTCACTA	GTGATAGCCTGGCGGTAACG
β-tubulin	CTCCAGCTCGAGCGTATGAAC	GGCTCCAAATCGACGAGAAC
PatK-P1	TCCCCATCTGCCTGTTGAAC	AAGCCTGAACGGGATGCAT
PatK-P2	ATCCCGTTGTCCGTGAGTGA	CTCCTCCACGAGGGTTCCTT
PatK-E1	ATCCCCAGCACCAGCTGATA	AGCTCAGGGTTGTGGTTTCCT
PatK-E2	GCGTGAAGGTCGTCCAAGAG	GGGCCTGACAGCAACAAAAG
PatK-E3	GAGCGCGTGGAGATCTTCA	TGCACGAGACTCTCCATGCT
PatK-E4	CGGAGCTCAAGGCGTATCTG	TCGGGTGACTCCACGTCAAT
PatG-P	TTTTGCAAGCGCACATATCC	TGATCGGGAGACCATCATGA
PatG-E1	CCATCATGGCGAAAATCGAT	GGCGCAGTGACAGAGAGAATG
PatG-E2	TGAATACGCCTGCACCACTCT	GCTGCGGTAGCCAGGTGTT
PatG-E3	AGGCAGCCCGGACATTCTA	GCTGTCAACCTGTCGCTTTG
PatL-P1	TATCACCAGCCTCCATTTGGA	CAGAATCCGGGACGCTACAC
PatL-P2	ATTGATGCAAATCGCATTCG	ACTCCAATTAAGCCCCAAAGG
PatL-P3	TGGGCAAGATTTGTAGGTTCGT	TGAATTCGCCACTTCGCTTT
PatL-E1	TCACACCTCGTTCGACTTTGC	TAATGAAAGCGTGCCATGCA
PatL-E2	CCCTGCTGAATACGGCACAT	TGACATGGGACAGCTGAATGA
PatL-E3	CGCTGGACCTCGTGAGTGAT	GGCAACGAACGAGGTTTGAT
